# Impact of Clinical and Socio-Demographic Factors on the Quality of Life in Romanian People with Epilepsy

**DOI:** 10.3390/healthcare10101909

**Published:** 2022-09-29

**Authors:** Ionut-Horia Cioriceanu, Dan-Alexandru Constantin, Luigi Geo Marceanu, Costin-Vlad Anastasiu, Andreea Nicoleta Serbanica, Liliana Rogozea

**Affiliations:** 1 Clinical Hospital of Psychiatry and Neurology Brasov, 500123 Brasov, Romania; 2 Department of Fundamental, Prophylactic and Clinical Sciences, Faculty of Medicine, Transilvania University of Brasov, 500019 Brasov, Romania; 3 Department of Medical and Surgical Specialties, Faculty of Medicine, Transilvania University of Brasov, 500019 Brasov, Romania; 4 Department of Pediatrics, Faculty of Medicine, Carol Davila University of Medicine and Pharmacy, 050474 Bucharest, Romania

**Keywords:** epilepsy, quality of life, QOLIE-31-P

## Abstract

This study investigates the impact of different clinical and demographic factors on the quality of life in people with epilepsy hospitalized at a health institution of Brasov County, Romania, using a QOLIE-31-P questionnaire and to reflect on the opportunities and limitations of incorporating such an instrument into the clinical practice. Methods: Ninety-one patients with a diagnosis of epilepsy evaluated by video-electroencephalography in the Clinical Hospital of Psychiatry and Neurology in Brasov, Romania, were recruited. After the confirmation of the diagnosis based on clinical, electrophysiological and imagistic examination, and of their compliance with the hospitalization criteria, the patients filled in the QOLIE-31-P questionnaire. Socio-demographic and clinical data were collected. Results: The seizure frequency was negatively correlated with almost all QOLIE-31-P domains (*p* < 0.05). Age, employment status, level of education and uncontrolled disease were significant factors associated with a low quality of life. The mean (SD) QOLIE-31-P scores were 64.89 (14.72), the mean age was 43.04 (14.92) years, with the average age of the first seizure onset 30.66 (17.45) years. Conclusion: The use of measuring instruments to assess the quality of life of patients with epilepsy despite the challenges should become a routine practice, the information collected in this way can improve the outcomes in the care of these patients. In addition to the goal of reducing the frequency of seizures, physicians must also take into account other parts of the experiences of people with epilepsy.

## 1. Introduction

Epilepsy is a brain disorder defined by recurrent seizures and the presence of these seizures has a major impact on quality of life (QOL). The concept of QOL is defined as an “individual’s perception of their position in life in the context of the culture and value systems in which they live and in relation to their goals, expectations, standards and concerns” [1]. Epilepsy can be controlled in a relatively large proportion of patients [2] and adequate therapy improves medical and social prognosis of patients with epilepsy (PWE) [3]. PWE with uncontrolled seizures may suffer from employment difficulties, educational and relationship problems in their lives due to social discrimination, stigmatization and fear of having seizures [4].

In addition to a clinical evaluation that must take into account a complete history, imaging and electroencephalographic investigations, evaluations are needed to identify how PWE perceive the QOL, factors influencing the perception and the opportunity to use measuring instruments in clinical routine. Obtaining and using this information can help in therapy planning with improvement in seizure control, anti-seizure medication (ASM) side effects and overall well-being of patients [5]. Using the QOLIE-31-P questionnaire as a routine in clinical practice depends on the physician’s determination to spend the time required to apply the questionnaire and to review the results during patient consultation or investigation.

Although there are numerous studies worldwide analyzing the QOL of PWE, in Romania we do not have enough data and there are no studies published in the scientific literature in the past years using this tool. This study aims to identify the effect of demographic and disease characteristics on the QOL of patients with epilepsy admitted at a Romanian county hospital through the QOLIE-31-P questionnaire and to reflect on the opportunities and limitations of incorporating such an instrument into clinical practice.

## 2. Material and Methods

### 2.1. Participants

This research included ninety-one patients aged 18–79 years with a diagnosis of epilepsy according to the International Classification of Epilepsies and Epileptic Syndromes (International League Against Epilepsy (ILAE)) criteria [6], admitted at the Clinical Hospital of Psychiatry and Neurology Brasov, Romania, for evaluation by video electroencephalography (VEEG). Between February 2018 and August 2021, the people who agreed to participate in the research were enrolled. No special selection was performed, only the patients with another progressive neurological or psychiatric disease, severe somatic pathology, intellectual disability or those who had difficulties in understanding the aspects of the instrument, were excluded.

### 2.2. Study Instruments

The Patient-Weighted Quality of Life in Epilepsy Inventory—QOLIE-31-P© [7] is developed for use for adults aged of 18 years and older, and it was created and modified from the original Quality of Life in Epilepsy Inventory (QOLIE-31) version 1 [8]. The QOLIE-31-P includes 30 items about health concepts, following seven multi-item scales: emotional well-being—5 items, social functioning—5 items, energy/fatigue—4 items, cognitive functioning—6 items, seizure worry—5 items, medication effects—3 items, and overall quality of life—2 items, and adds one new item into each subscale asking about the level of distress, defined as bothersomeness, for the respondent associated with the topic of each subscale. The scoring procedure is in accordance with the Scoring Manual for the QOLIE-31-P and the total and subscales scores values range from 0 to 100, with higher scores reflecting greater QOL. The scoring procedure first converts the answers of items to 0–100-point scores, with higher scores reflecting greater well-being. The QOLIE-31-P subscale scores are the means of the converted item scores multiplied by its distress item. The total score is calculated by averaging the subscale weighted scores [9]. Before applying it to the participants, we asked for permission and copyright. Cramer J. provided and returned the Romanian version of QOLIE-31-P, which we used in this study.

### 2.3. Procedures

The patients with the diagnosis of epilepsy who presented themselves for the VEEG investigation were identified as their documents for admission were brought to the VEEG unit. After confirmation of the diagnosis based on clinical, electrophysiologic and imagistic examinations, the patients were invited, and the aim of the research was described to them. They were given the opportunity to ask questions about the research and it was explained to them that no inconvenience would incur from a refusal to participate. Those who agreed signed consent forms to participate in the research and for the publication of the findings. Socio-demographic data, such as age, gender, living environment, marital status, employment status, and level of education, were collected. It also included the following clinical data on epilepsy: age of onset, frequency of seizures, epilepsy type by onset, presence of aura, etiology, type of seizures, presence of seizures in sleep, number of ASM and last episode of seizures. The questionnaires were then administered, allocating numbers to accomplish confidentiality.

Digital VEEG was performed, and the location of epileptiform activity (EA) was studied.

### 2.4. Data Analysis

The data were analyzed by means of the GraphPad Prism version 9.2.0 using absolute values and percentages for categorical variables and processing by methods of variations statistics, including measuring of mean scores, standard deviation (SD) and other parameters. The last seizure was used to divide the seizure control into two groups: controlled group, who had no seizures in the past year, and uncontrolled group, who had at least one seizure in the past year. QOLIE-31-P total scores (TS) were classified using mean scores into three groups: participants whose score was ≥1 SD from the mean were classified as having high QOL, those whose score was within 1 SD were classified as average and those with a score ≤ 1 SD were classified as having low QOL. For testing the associations between the socio-demographic and clinical variables with the QOLIE-31-P TS and subscales scores, independent *t*-test for 2 groups and Analysis of Variance (ANOVA) for >2 groups were used for quantitative variables; Chi-2 test was used for the qualitative variables. Significant variables found in the univariate analysis were introduced into a multiple linear regression model for testing predictors of the QOLIE-31-P TS and subscale scores. A *p*-value of <0.05 and a confidence interval of 95% were set as level of statistical significance in all tests.

### 2.5. Ethical Approval

Before the study began it was approved by the Research and Ethics Committee of Transilvania University of Brasov and by the Director of the Clinical Hospital of Psychiatry and Neurology Brasov, Romania.

## 3. Results

From the total of 91 PWE in this study, 57.1% (*n* = 52) were females. The mean (SD) age was 43.04 (14.92) years. Seventy-five-point-eight percent (*n* = 69) of participants lived in urban areas, 62.6% (*n* = 57) were married and 45.1% (*n* = 41) were employed. Most participants, 46.2% (*n* = 42), had a high formal level of education. The mean (SD) age of onset of epilepsy was 30.66 (17.75) years, 70.3% (*n* = 64) with onset after the age of 18 and the mean (SD), duration of epilepsy was found to be 12.38 (14.90) years. According to the ILAE criteria, structural epilepsy was characterized in 52.7% (*n* = 48) of cases, genetic in 4.4% (*n* = 4) of cases and unknown in 42.9% (*n* = 39) of cases. The seizure type was divided by onset into focal seizure in 86.8% (*n* = 79) and generalized seizure in 13.2% (*n* = 12) of the cases; 85.7% (*n* = 78) had bilateral tonic–clonic seizures, 26.4% (*n* = 24) experienced frequent seizures defined as two or more/month. The majority of the patients were on monotherapy and had no aura preceding seizures (Table 1).

Mean QOLIE-31-P TS of the respondents was 64.89 (±14.72). For the energy domain the score was 31.67 (±27.28), for mood 35.37 (±25.79), for the daily activities domain 43.32 (±32.17), for cognition 46.32 (±33.74), for medication effects 46.83 (±34.06), for the seizure worry domain 28.89 (±32.05) and for the overall QOL domain 37.91 (±25.54). Twenty-three-point-one percent (*n* = 21) of patients had low QOL, 49.5% (*n* = 45) had average and 27.5% (*n* = 25) had high QOL. The correlation of QOL scores across the domains are shown in Table 2. Comparing the two age groups, patients aged 44–79 years had lower scores on the energy, mood, cognition, seizure worry and overall QOL domains. Compared with males, females had lower scores on the mood domain. Being ill-health retired was associated with poor scores on the mood, daily activities, cognition, seizure worry and overall QOL domains. Patients with elementary school (4 years of study) and those with middle school (8 years of study) had low scores in overall QOL domain. Seizure frequency was negatively correlated with almost all QOLIE-31-P domains, except for the medication effects domain; those who had two or more seizures per month reported the lowest values in energy, mood, daily activities, cognition and seizure worry domains. Uncontrolled seizures predicted lower scores in mood, daily activities, cognition, seizure worry and overall QOLIE domains. These results are supported by statistically significant values of *p* less than 0.05.

Following multiple regression, the predictors of cognition (*t* = 3.743, *p* = 0.0003) and medication effects (*t* = 3.481, *p* = 0.0008) had the most important influence on the QOLIE-31-P total score (TS).

## 4. Discussion

In this study the mean QOLIE-31-P TS for the patients enrolled was lower than studies conducted in Greece [10], the Czech Republic [11] and the United Kingdom [12] but higher than in research made in Bulgaria [13], Russia [14], France [15], Germany [16] and Spain [17]. It was also higher compared with the European and global QOLIE-31 scores [18] and a major part of the patients enrolled had an average QOL when the scores were categorized. Scores may be different due to health conditions and demographic factors, such as age [19], gender [20] or level of education [21] and higher scores could represent better disease information and awareness, social support and decrease in stigma.

This study demonstrates that the patient’s age has a negative impact on the energy, mood, cognition, seizure worry, overall QOL domains and QOLIE-31-P TS, similar to the correlations reported in other studies [22] and the higher the age of the patient is, the lower his/her quality of life is. Contradictory results show no significant correlations between age and total score [23].

Females had significantly lower scores on the mood domain and this may indicate that both biological and psychological factors, such as personal life or motherhood, may have a more important role than socioeconomic factors, in accordance with other Romanian research performed in the past [24].

It is well documented that PWE have lower education and income and finding a job is more difficult for them [25]. The level of education is one of the important predictors of the quality of life [26] and this study showed that overall QOL score and QOLIE-31-P TS were significantly influenced by the years of study. Compared with a study that included European countries where the employment rate altered all QOLIE domains [27], in this study statistical analysis showed that ill-retired and retired participants had lower scores of mood, daily activities, cognition, seizure worry, overall QOL domains and QOLIE-31-P TS than those who were employed. This may be influenced by poor economic status, as these categories have low incomes compared to those employed or other European citizens [28].

Across all domains, except medication effects, the increase in seizure frequency was a predictor of lower QOL scores. Additionally, patients with uncontrolled seizures reported lower QOL on mood, daily activities, cognition, seizure worry, overall quality of life and QOLIE-31-P TS. Because uncontrolled seizures were found to impact the patient’s family, work and social life [29], if physicians can reduce the frequency of seizures by adequate evaluation and treatment, they may improve the QOL of PWE. In addition to the goal of reducing the frequency of seizures, the physicians must also take into account other parts of the experiences of patients with epilepsy.

The least affected domain was the medication effects domain, one of the predictors with the maximum influence on the QOLIE-31-P TS. Most patients were on ASM monotherapy which reduces the probability of adverse effects and increase the confidence in treatment. Side effects are associated with number of ASMs and had been correlated with lower QOL scores in other studies [30].

This study included participants with a diagnosis of epilepsy evaluated by VEEG and the location of epileptiform activity was also studied. There were no uncertainties regarding the diagnosis, type of seizures or epileptic syndrome, but also there were no statistically significant correlations between epileptiform activity, seizures onset, etiology, presence of aura, seizures type, presence of seizures in sleep and QOLIE-31-TS and domain scores. In previous studies of QOL in PWE that provided these details is evidence that the type of seizure is a predictor of QOL. It was demonstrated that both focal and generalized seizures negatively affected all domains of QOL [31] despite that in other study seizure type was an insignificant predictor [32]. Future studies should investigate the QOL of PWE while including details of seizure types in their analysis to identify potential predictors. Seizure freedom is the primary objective of treatment, but for some PWE this is not possible. Understanding predictors of QOL could improve clinical practice and treatment for PWE and support them in fulfill desired outcomes.

Validated instruments were not used to assess the presence of depressive symptoms because we only document this when PWE are suspected of having symptoms and we seek further consultation from a psychologist.

The goal of the tools for measuring the quality of life of patients with epilepsy is to support the physician in making decisions about the treatment and management of these patients. Dividing the QOLIE-31-P total scores in this study into low QOL, medium QOL and high QOL using the mean scores was attempted to support the physician in identifying patients with poor QOL, in looking for the factors that contribute thereto, and then in taking measures for improving the quality of life of these patients.

Scoring the QOLIE-31-P questionnaire can be challenging in routine use because it uses a weighted scale score which can be difficult to process immediately after administration [33]. In order to streamline the whole procedure, the questionnaire could be administered after the patient’s anamnesis, before the preparation for the VEEG recording, and the processing could be performed by the physician during the recording, using a template in electronic format. Once the interpretation of the investigation is released, the patient will receive recommendations also based on the QOL score, such as advice for psychological support for patients with low QOL scores and signs of anxiety [34], adaptation to different workplaces or treatment decisions.

In this way, the patient would not wait too long which would increase his/her burden, the nurse would not be overloaded with another task, and the results of the investigation and the QOLIE-31-P scores can be recorded in the hospital software and may be used in the future for reassessment and comparison. Moreover, patients can be more actively involved in the management of their disease and better supported through self-management training programs. These programs use the theoretical model of behavioral change that includes strategies for improving the relevant knowledge, skills and self-sufficiency and can improve QOL [35].

The most important barrier to this study was the lower access of patients with epilepsy to assessments, which occurred after the outbreak of the COVID-19 pandemic. First discovered in December 2019, COVID-19, caused by SARS-CoV-2, was declared an epidemic by the WHO on 11 March 2020 and within months it had extended to the whole world [36]. From April 2020 the hospital where the study was conducted was included on the list of national hospitals for the treatment of patients infected with the SARS-CoV-2 virus; and the VEEG monitoring unit, located in one of the hospital departments, could not be moved, which led to the blockage of its use, when there were infected patients hospitalized in that area. Therefore, the PWE evaluations were postponed, and they could not go to other public hospitals because the VEEG unit is the only one in town. A study conducted during the COVID-19 pandemic also demonstrated the healthcare availability issues and perceived fears with negative impact on the lives of patients with epilepsy [37].

Future studies with participants from different regions of the country are needed to obtain more accurate results and a comparison of the QOL of PWE before, during and after the COVID-19 pandemic would be of interest.

## 5. Conclusions

Until now in our country there is a lack of studies on the quality of life for patients with epilepsy.

This research has shown that seizure frequency has an important impact on the QOL of PWE. The use of measuring validated instruments to assess the QOL of PWE, such as the QOLIE-31-P questionnaire, should become a routine clinical practice even if this may be challenging. The information collected in this way can tailor the management and improve the outcome for these patients by looking for the influence of disease and other modifiable factors in everyday life.

## Figures and Tables

**Table 1:** Socio-demographic and clinical characteristics of participants and QOLIE-31-P TS.

Variables	QOLIE-31-P TS Classification *			QOLIE-31-P TS **
	Low *n* (%)	Average *n* (%)	High *n* (%)	Mean (SD)
**Age**				
18–44	9 (17.6)	34 (66.7)	8 (15.7)	69.56 (15.30)
45–79	6 (15.0)	28 (70.0)	6 (15.0)	58.93 (18.97)
*p*-value	0.9330			**0.0039**
**Sex**				
Male	6 (15.4)	19 (48.7)	14 (35.9)	64.14 (16.24)
Female	10 (19.2)	38 (73.1)	4 (7.7)	65.52 (18.91)
*p*-value	**0.0036**			0.7155
**Environment**				
Urban	10 (14.5)	49 (71.0)	10 (14.5)	65.89 (18.25)
Rural	5 (22.7)	12 (54.6)	5 (22.7)	61.73 (15.94)
*p*-value	0.3593			0.3406
**Marital status**				
Single	5 (14.7)	26 (76.5)	3 (8.8)	67.49 (17.84)
Married	10 (17.5)	38 (66.7)	9 (15.8)	63.34 (17.63)
*p*-value	0.5547			0.2824
**Employment status**				
Without job	4 (25.0)	10 (62.5)	2 (12.5)	64.69 (17.72)
With job	6 (14.6)	29 (70.8)	6 (14.6)	72.79 (13.65)
Student	1 (25.0)	2 (50.0)	1 (25.0)	68.38 (10.45)
Ill-health retired	3 (33.3)	5 (55.6)	1 (11.1)	44.91 (16.66)
Retired	3 (14.3)	15 (71.4)	3 (14.3)	57.51 (17.63)
*p*-value	0.9340			**0.0001**
**Level of education**				
Primary	1 (33.3)	2 (66.7)	0 (0.0)	36.47 (4.865)
Secondary	2 (16.7)	8 (66.6)	2 (16.7)	58.56 (13.44)
High-school	7 (16.7)	29 (69.0)	6 (14.3)	66.15 (18.59)
Technical-school	2 (11.8)	13 (76.4)	2 (11.8)	64.04 (18.73)
Higher	2 (11.8)	12 (70.6)	3 (17.6)	72.10 (12.71)
*p*-value	0.9867			**0.0123**
**Age of onset (years)**				
<18	4 (14.8)	18 (66.7)	5 (18.5)	61.14 (19.88)
>18	14 (21.9)	41 (64.0)	9 (14.1)	66.47 (16.65)
*p*-value	0.6895			0.1917
**Seizure frequency**				
1/month	1 (16.7)	4 (66.6)	1 (16.7)	52.98 (19.78)
2 or >2/month	4 (16.7)	16 (66.6)	4 (16.7)	50.11 (15.00)
1–6/year	10 (19.2)	35 (67.3)	7 (13.5)	70.54 (14.82)
No seizure in the last year	1 (11.1)	7 (77.8)	1 (11.1)	79.57 (5.5)
*p*-value	0.9403			**<0.0001**
**Epileptiform activity**				
Right	4 (13.3)	21 (70.0)	5 (16.7)	62.49 (18.92)
Left	3 (13.6)	15 (68.2)	4 (18.2)	61.83 (17.78)
Bilateral	4 (13.3)	21 (70.0)	5 (16.7)	68.48 (16.51)
Without	1 (11.1)	7 (77.8)	1 (11.1)	68.36 (17.55)
*p*-value	0.9994			0.4322
**Number of ASM taken**				
Monotherapy	11 (21.6)	34 (66.7)	6 (11.8)	69.11 (17.47)
≥2	4 (15.4)	16 (61.5)	6 (23.1)	52.81 (15.50)
Without	3 (21.4)	9 (64.3)	2 (14.3)	72.21 (11.04)
*p*-value	0.7593			**<0.0001**
**Epilepsy type (onset)**				
Focal	15 (19.0)	50 (63.3)	14 (17.7)	64.26 (17.59)
Generalized	1 (8.3)	11 (91.7)	0 (0.0)	69.00 (18.80)
*p*-value	0.1320			0.3909
**Etiology**				
Unknown	9 (23.1)	26 (66.7)	4 (10.3)	68.16 (17.57)
Structural	10 (20.8)	28 (58.3)	10 (20.8)	63.37 (17.07)
Genetic	1 (25.0)	3 (75.0)	0 (0.0)	52.06 (23.70)
*p*-value	0.6241			0.1519
**Presence of aura**				
Yes	5 (14.2)	22 (62.9)	8 (22.9)	61.12 (17.64)
No	11 (19.6)	37 (66.1)	8 (14.3)	67.24 (17.52)
*p*-value	0.5257			0.1094
**Seizure type**				
With motor tonic–clonic	15 (19.2)	51 (65.4)	12 (15.4)	65.01 (18.51)
Without motor tonic–clonic	3 (23.1)	7 (53.8)	3 (23.1)	64.13 (12.48)
*p*-value	0.6990			0.8694
**Seizures in sleep**				
Yes	4 (23.5)	12 (70.6)	1 (5.9)	72.09 (16.47)
No	12 (16.2)	51 (68.9)	11 (14.9)	63.23 (17.69)
*p*-value	0.5294			0.0627
**Seizure control**				
Uncontrolled	15 (18.3)	51 (62.2)	16 (19.5)	63.28 (17.87)
Controlled	1 (11.1)	7 (77.8)	1 (11.1)	79.57 (5.55)
*p*-value	0.6527			**0.0081**

* Chi-2 test. ** Independent *t*-test for 2 groups and ANOVA for >2 groups. The bold *p*-values (<0.05) represent significant difference between groups with regards to a domain score.

**Table 2:** Socio-demographic, clinical features and QOLIE-31-P domains.

Variables	*n*	Energy	Mood	Daily Activities	Cognition	Medication Effects	Seizure Worry	Overall Quality of Life
**Total** **(Mean, SD)**	91	31.67 (27.28)	35.37 (25.79)	43.42 (32.17)	46.32 (33.74)	46.83 (34.06)	28.89 (32.05)	37.91 (25.54)
Age *								
18–44	51	37.75 (28.75)	40.00 (26.72)	47.83 (32.71)	53.45 (33.52)	52.81 (34.25)	35.44 (33.58)	44.96 (25.21)
45–79	40	23.91 (23.41)	29.47 (23.58)	37.81 (30.96)	37.24 (32.20)	39.19 (32.65)	20.53 (28.24)	28.93 (23.28)
*p*-value		**0.0155**	**0.0224**	0.1412	**0.0221**	0.0579	**0.0268**	**0.0025**
Sex *								
Male	39	37.12 (29.11)	43.55 (28.31)	46.38 (34.12)	49.46 (33.67)	49.91 (33.72)	36.47 (35.74)	40.34 (26.46)
Female	52	27.58 (25.35)	29.23 (22.08)	41.20 (30.77)	43.96 (33.94)	44.51 (34.45)	23.20 (28.01)	36.09 (24.92)
*p*-value		0.0991	**0.0080**	0.4502	0.4448	0.4572	0.0501	0.4351
Environment *								
Urban	69	29.59 (25.79)	36.77 (26.79)	42.86 (32.76)	43.44 (32.99)	45.99 (34.40)	30.23 (33.57)	37.17 (25.28)
Rural	22	38.19 (31.27)	30.98 (22.36)	45.19 (30.91)	55.37 (35.24)	49.45 (33.59)	24.70 (27.03)	40.23 (26.80)
*p*-value		0.1996	0.3621	0.7692	0.1497	0.6805	0.4841	0.6272
Marital status *								
Single	34	29.07 (24.40)	35.81 (25.18)	40.07 (31.64)	44.94 (34.34)	41.05 (35.55)	33.35 (34.27)	38.71 (30.44)
Married	57	33.21 (28.97)	35.11 (26.37)	45.42 (32.59)	47.15 (33.66)	50.27 (32.97)	26.23 (30.66)	37.43 (22.39)
*p*-value		0.4869	0.9012	0.4458	0.7643	0.2134	0.3080	0.8185
Employment status **								
Without job	16	23.98 (19.67)	37.50 (24.43)	43.34 (38.40)	50.25 (38.45)	55.38 (33.34)	25.91 (30.66)	36.28 (25.79)
With job	41	39.01 (29.12)	46.57 (28.45)	54.79 (28.94)	59.03 (32.79)	48.96 (31.81)	40.44 (34.15)	48.80 (24.25)
Student	4	34.38 (41.31)	19.85 (24.83)	49.38 (20.87)	36.89 (36.44)	60.07 (30.37)	15.89 (13.65)	56.09 (30.39)
Ill-health retired	9	17.61 (11.75)	17.58 (11.89)	16.47 (21.13)	25.53 (24.53)	24.25 (31.49)	5.02 (7.97)	22.32 (20.73)
Retired	21	24.05 (19.51)	27.10 (18.93)	30.95 (29.28)	34.06 (29.29)	44.85 (35.78)	17.38 (26.58)	22.36 (17.61)
*p*-value		0.0529	**0.0031**	**0.0036**	**0.0136**	0.1964	**0.0051**	**0.0002**
Level of education **								
Primary	3	11.33 (14.11)	14.00 (11.14)	3.23 (2.54)	6.32 (3.27)	2.82 (3.17)	3.45 (3.44)	6.21 (3.32)
Secondary	12	22.96 (25.49)	26.92 (24.71)	32.87 (30.73)	36.50 (18.41)	54.06 (35.93)	22.03 (30.51)	26.93 (22.40)
High-school	42	36.05 (29.67)	37.38 (26.77)	44.63 (30.29)	47.16 (33.87)	49.34 (34.61)	29.33 (32.83)	41.17 (23.60)
Technical-school	17	30.51 (26.63)	33.53 (22.73)	49.65 (35.11)	48.36 (36.80)	51.89 (32.17)	28.93 (32.01)	33.40 (28.07)
Higher	17	31.72 (23.74)	42.00 (27.45)	48.75 (33.53)	56.20 (37.34)	43.67 (30.56)	37.10 (33.51)	47.72 (25.99)
*p*-value		0.4116	0.3106	0.1262	0.1462	0.1727	0.4759	**0.0281**
Age of onset (years) *								
<18	27	30.57 (28.20)	33.59 (26.04)	43.02 (33.42)	48.99 (36.11)	48.48 (31.88)	25.65 (31.85)	33.76 (25.91
>18	64	32.13 (27.10)	36.12 (25.86)	43.59 (31.89)	45.19 (32.93)	47.62 (34.70)	30.25 (32.29)	39.66 (25.38)
*p*-value		0.8048	0.6715	0.9390	0.6263	0.9122	0.5347	0.3168
Seizure frequency **								
1/month	6	26.58 (28.78)	22.87 (15.63)	33.00 (36.39)	32.16 (24.69)	25.37 (23.05)	10.55 (10.71)	18.65 (7.95)
2 or >2/month	24	15.78 (10.79)	21.60 (18.40)	22.90 (22.76)	24.01 (24.39)	36.87 (34.11)	7.36 (10.15)	21.56 (17.13)
1–6/year	47	33.96 (27.36)	35.27 (23.15)	46.51 (30.52)	50.51 (33.04)	52.58 (35.11)	36.36 (34.90)	42.19 (25.48)
No seizures in the last year	14	31.38 (28.32)	38.50 (26.93)	46.93 (30.93)	54.80 (36.72)	52.93 (31.67)	30.09 (30.90)	42.79 (25.50)
*p*-value		**0.0332**	**0.0451**	**0.0114**	**0.0039**	0.1047	**0.0008**	**0.0010**
Epileptiform activity **								
Right	30	29.78 (25.88)	29.85 (20.76)	29.62 (24.80)	38.78 (34.44)	49.50 (35.19)	24.44 (28.50)	34.74 (27.26)
Left	22	23.69 (23.86)	34.44 (30.89)	40.30 (32.43)	41.73 (32.44)	35.67 (29.09)	21.23 (23.29)	35.23 (22.98)
Bilateral	30	34.27 (27.62)	38.07 (25.09)	53.49 (33.34)	51.45 (33.24)	47.66 (34.28)	31.52 (37.55)	42.33 (27.15)
Without	9	45.97 (24.81)	41.56 (24.52)	54.58 (40.17)	63.47 (21.26)	42.74 (35.09)	24.25 (27.64)	35.28 (22.36)
*p*-value		0.1609	0.5158	**0.0209**	0.1602	0.4832	0.6587	0.6544
Number of ASM taken **								
Monotherapy	51	29.10 (27.54)	31.89 (26.72)	44.60 (31.77)	47.30 (33.90)	47.49 (32.87)	26.40 (32.17)	39.07 (26.54)
≥2	26	34.00 (26.46)	37.55 (24.21)	36.95 (31.03)	40.50 (33.01)	43.35 (36.03)	29.30 (35.26)	36.13 (25.25)
Without	14	36.38 (28.76)	44.00 (24.40)	51.13 (35.73)	53.56 (35.20)	50.84 (36.53)	37.21 (25.34)	36.99 (23.80)
*p*-value		0.5939	0.2643	0.3863	0.4867	0.7885	0.5390	0.8849
Epilepsy type (onset) *								
Focal	79	32.21 (27.16)	35.77 (24.92)	43.19 (32.77)	46.66 (34.50)	48.57 (34.86)	29.34 (32.44)	37.91 (25.88)
Generalized	12	28.13 (29.06)	32.77 (32.11)	44.94 (29.14)	44.11 (29.51)	35.37 (26.64)	25.91 (30.58)	37.93 (24.17)
*p*-value		0.6320	0.7096	0.8618	0.8089	0.2128	0.7319	0.9980
Etiology **								
Unknown	39	29.10 (24.44)	34.94 (24.45)	45.73 (31.87)	43.92 (33.90)	51.93 (35.66)	31.37 (35.32)	43.55 (27.22)
Structural	48	32.86 (25.88)	35.65 (25.05)	43.91 (30.64)	47.30 (34.06)	51.98 (33.54)	31.46 (33.00)	37.79 (24.84)
Genetic	4	30.63 (31.84)	40.50 (39.08)	45.50 (34.82)	43.79 (39.98)	58.68 (41.55)	36.25 (44.16)	47.81 (34.93)
*p*-value		0.7912	0.9163	0.9632	0.8946	0.9310	0.9631	0.5145
Presence of aura *								
Yes	35	29.14 (24.87)	31.27 (24.33)	35.36 (26.93)	44.32 (32.33)	42.68 (31.87)	22.71 (29.49)	33.52 (20.32)
No	56	33.25 (28.80)	37.93 (26.56)	48.46 (34.32)	47.57 (34.83)	49.42 (35.39)	32.76 (33.23)	40.65 (28.14)
*p*-value		0.4876	0.2328	0.0583	0.6574	0.3613	0.1466	0.1967
Seizure type *								
With motor tonic–clonic	78	33.65 (28.45)	35.75 (26.20)	42.90 (32.63)	47.74 (35.20)	47.91 (35.43)	27.91 (31.88)	37.69 (26.88)
Without motor tonic–clonic	13	19.79 (14.55)	33.11 (24.02)	46.57 (30.29)	37.82 (22.30)	40.33 (24.32)	34.80 (33.78)	39.23 (15.86)
*p*-value		0.0901	0.7346	0.7056	0.3292	0.4606	0.4761	0.8417
Presence of seizures in sleep *								
Yes	17	41.60 (30.35)	47.12 (29.28)	53.34 (33.68)	59.51 (30.44)	49.20 (36.30)	40.22 (35.43)	45.26 (28.41)
No	74	29.39 (26.22)	32.67 (24.32)	41.14 (31.60)	43.29 (33.93)	46.28 (33.76)	26.29 (30.90)	36.22 (24.73)
*p*-value		0.0963	**0.0364**	0.1596	0.0738	0.7519	0.1065	0.1897
Seizure control *								
Uncontrolled	82	30.22 (27.08)	32.62 (25.08)	40.08 (31.05)	43.86 (33.60)	46.34 (34.11)	26.57 (31.04)	35.07 (24.43)
Controlled	9	44.86 (27.07)	60.44 (18.16)	73.83 (26.92)	68.73 (27.47)	61.58 (27.62)	50.04 (35.30)	63.75 (21.39)
*p*-value		0.1272	**0.0017**	**0.0024**	**0.0351**	0.1995	**0.0363**	**0.0011**

* Independent *t*-test. ** ANOVA.

## Data Availability

The data presented in this study are available on request from the corresponding author.
